# Инвазивный рост аденом гипофиза: значение матриксных металлопротеиназ

**DOI:** 10.14341/probl13631

**Published:** 2026-03-07

**Authors:** Д. В. Кутакова, А. С. Луценко, Е. Г. Пржиялковская, В. Н. Азизян

**Affiliations:** Национальный медицинский исследовательский центр эндокринологии им. И.И. ДедоваРоссия; Endocrinology Research CentreRussian Federation

**Keywords:** инвазивные аденомы гипофиза, матриксные металлопротеиназы (ММП), ММП-2, ММП-9, invasive pituitary adenoma, matrix metalloproteinase (MMP), MMP-2, MMP-9

## Abstract

Аденомы гипофиза (АГ) являются наиболее часто встречаемыми опухолями гипоталамо-гипофизарной области. Клиническая картина АГ зависит как от гормональной активности, так и от особенностей распространения опухоли. Несмотря на то, что аденомы гипофиза в подавляющем большинстве случаев являются доброкачественными, они могут расти инвазивно и механически воздействовать на прилежащие структуры. При инвазивном росте аденом гипофиза радикальное удаление затруднительно и сопряжено с более высоким риском операционных осложнений. Патогенез инвазивности аденом гипофиза полностью не изучен. Инвазия опухолевых клеток зависит как от межклеточных взаимодействий внутри аденомы, так и от связи с компонентами внеклеточного матрикса (ВКМ). К факторам, играющим важную роль в данных процессах, относятся ферменты из семейства матриксных металлопротеиназ (ММП) и тканевые ингибиторы матриксных металлопротеиназ (ТИМП). Наибольшее внимание в отношении инвазивности аденом гипофиза уделяется двум представителям ММП — 2 и 9 типов. Интерес к данным молекулам обусловлен их участием в деградации коллагена IV типа, являющегося ключевым компонентом ВКМ гипоталамо-гипофизарной области. В данном обзоре обсуждается общая концепция инвазивности аденом гипофиза, характеристика ММП и исследования, посвященные взаимосвязи данных молекул с инвазией аденом гипофиза.

## Abstract (en)

Pituitary adenomas (PA) are the most common lesions of hypothalamic-pituitary region. Clinical presentations of PA depend on both hormonal activity and tumor growth characteristics. Despite the fact that PA are mostly benign, they can grow invasively and mechanically affect adjacent structures. With invasive growth of PA, radical removal is difficult and associated with a higher risk of surgical complications. Pathogenesis of PA invasiveness is not fully understood. Tumor cell invasion depends on both intercellular interactions within the tumor and interaction with extracellular matrix components (ECM). Major factors that play an important role in these processes include matrix metalloproteinase (MMP) family and tissue inhibitors of matrix metalloproteinases (TIMPs). Two MMPs were mostly studied in PA – types 2 and 9. These molecules are of interest, as they participate in degradation of type IV collagen, which is a key component of the ECM in hypothalamic-pituitary region. This review discusses the general concept of PA invasiveness, the characteristics of MMPs, and research of the relationship between these molecules and PA invasiveness.

## Введение

Опухоли гипофиза встречаются часто, их доля среди всех внутричерепных новообразований составляет около 10–25% [1]. Более 90% опухолей гипофиза являются доброкачественными аденомами [2], по различным эпидемиологическим данным их распространенность cоставляет около 80–90 на 100 тыс. населения [3]. По размеру аденомы гипофиза (АГ) принято разделять на микроаденомы (<10 мм в диаметре), макроаденомы (≥10 мм в диаметре) и гигантские аденомы (диаметром >60 мм).

Клиническая картина АГ может сильно различаться и зависит как от гормональной активности, так и механического воздействия образования на прилежащие структуры [4]. Несмотря на доброкачественную природу, АГ могут расти инвазивно, вовлекая диафрагму турецкого седла, супраселлярную цистерну, хиазму, третий желудочек головного мозга, твердую мозговую оболочку, дно турецкого седла, решетчатый лабиринт, кавернозные синусы и т.д. [5]. Согласно патоморфологическому исследованию 2002 г., из представленных 354 образов АГ около 45% опухолей имели признаки инвазии в твердую мозговую оболочку [6].

Нейрохирургическое лечение является основным методом лечения АГ, за исключением пролактин-секретирующих. Однако при инвазивном росте образования его радикальное удаление может быть затруднительным в связи с повышенным риском операционных осложнений: гипопитуитаризма, ликвореи, повреждения черепных нервов и внутренних сонных артерий [7].

Патогенез инвазивности аденом гипофиза на сегодняшний день окончательно не исследован. Предполагается, что косвенными признаками инвазии являются не только изменение состава внеклеточного матрикса (ВКМ), но и активность его компонентов. ВКМ обеспечивает механическую поддержку клеток и принимает активное участие в регуляции клеточного цикла, подвижности и передачи межклеточных сигналов. В состав ВКМ входят протеогликаны, гликозаминогликаны, структурные белки (коллаген и эластин), белки адгезии (фибронектин и ламинин) и протеолитические ферменты, также называемые матриксными металлопротеазами (ММП) [8]. Наибольшее внимание в отношении инвазивности АГ уделяется двум представителям ММП — 2 и 9 типам, так как они участвуют в метаболизме коллагена IV типа — ключевого компонента ВКМ гипоталамо-гипофизарной области. Кроме того, ММП-2 и ММП-9 потенциально могут использоваться в качестве биомаркеров инвазии, агрессивного роста и точек приложения для лечения аденом гипофиза.

Биомаркеры, связанные с ростом и/или инвазией опухоли, могут помочь определить агрессивность выявленного образования. Что, в свою очередь, позволит клиницистам заблаговременно определять наиболее подходящую тактику лечения и наблюдения. В данном обзоре обсуждается общая концепция инвазивности аденом гипофиза, характеристика ММП и исследования, посвященные взаимосвязи данных молекул с инвазией аденом гипофиза.

## Методы оценки инвазивности Аденом гипофиза

## Лучевой метод

Лучевые методы диагностики являются ключевым этапом обследования пациентов с заболеваниями гипоталамо-гипофизарной области, для того чтобы оценить размеры и степень распространения объемных образований. Как правило, инвазивность опухоли прямо пропорциональна ее размерам [9]. Согласно литературным данным, доля инвазивных аденом среди всех АГ варьирует от 2–3% [10] до 21% [11][12]. Не обнаружено четких взаимосвязей между инвазивностью АГ и половозрастными характеристиками. Однако считается, что наибольшим потенциалом инвазивности обладают молчащие кортикотропиномы, реже — опухоли, секретирующие тиреотропный гормон (ТТГ), соматотропный гормон (СТГ), пролактин (ПРЛ), адренокортикотропный гормон (АКТГ), фолликулостимулирующий гормон/лютеинизирующий гормон (ФСГ/ЛГ) и нуль-клеточные аденомы [13].

Самой широко используемой классификацией инвазии АГ является Knosp, определяющая степень инвазии в кавернозный синус относительно трех параллельных линий (медиальная, межсонная (средняя) и латеральная), проведенных между супраклиновидным и интракавернозным отделами внутренних сонных артерий (рис. 1а, 1с). Если опухоль распространяется за латеральную линию (Knosp 3) и полностью охватывает кавернозный сегмент внутренней сонной артерии (Knosp 4) — она относится к инвазивным (рис. 1б). При 3А степени опухоль распространяется в верхний латеральный отдел кавернозного синуса, тогда как при 3В опухоль охватывает нижний [15]. При степени 3А аденомы имеют более низкую скорость инвазии в сравнении с 3В и 4 [16]. Данный вывод был сделан на основании хирургической визуализации, при помощи эндоскопического оборудования: возможность интраоперационно исследовать зону медиальной стенки кавернозного синуса, находящейся за пещеристым сегментом ВСА, позволяет дифференцировать смещение тканей от инвазии аденомой гипофиза. Так, во многих исследованиях АГ 3А степени создавали «естественный коридор» для распространения ткани опухоли в кавернозный синус без его прорастания [17]. Следует отметить, что нет однозначных данных, свидетельствующих об отсутствии инфильтративного потенциала аденом со степенью Knosp≤2 [18].

**Figure:**
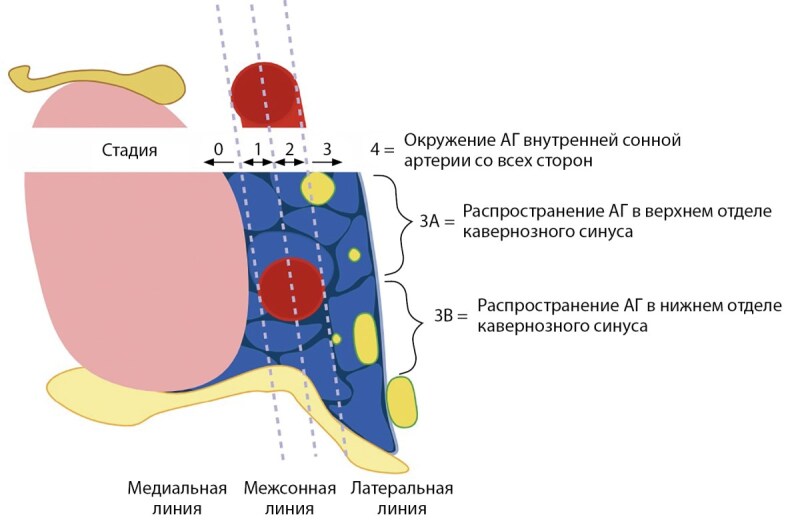
Рисунок 1а. Классификации инвазии аденом гипофиза по Knosp.

**Figure:**
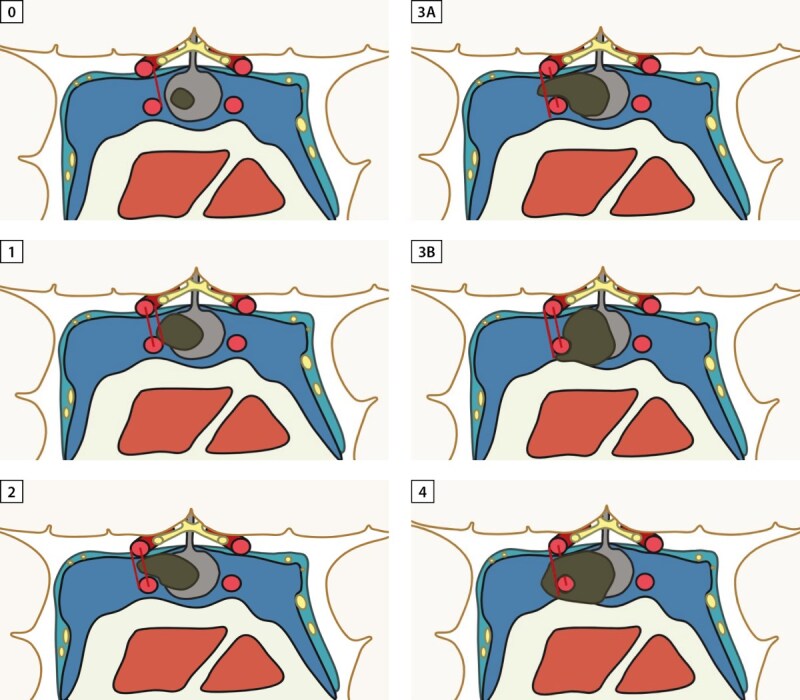
Рисунок 1б. Классификации инвазии аденом гипофиза по Knosp [14].

**Figure:**
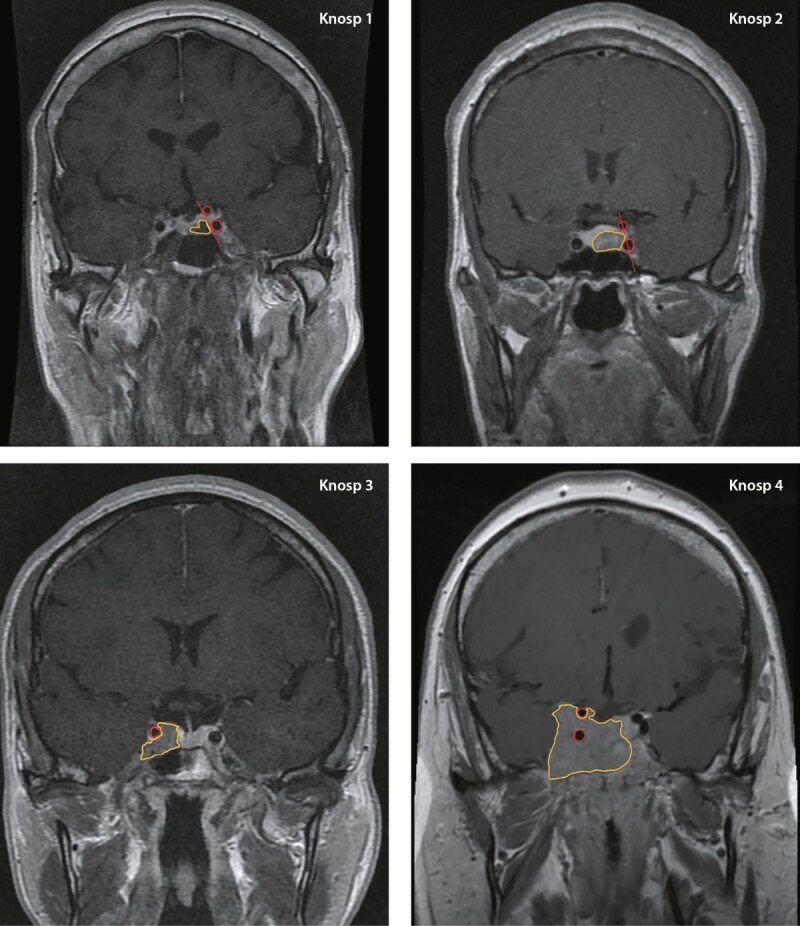
Рисунок 1с. Представление классификации Knosp на клинических примерах.

## Интраоперационный метод

В 1993 г. Knosp и соавт. [20] выделили четыре отдела кавернозного синуса относительно внутренней сонной артерии: медиальный, латеральный, верхний и нижний. Подтверждение хирургической инвазии было правомочно при сдавлении трех и более венозных отделов или при изолированном поражении латерального отдела. В 2017 г. Fernandez-Miranda и соавт. [21] предложили иное наименование отделов: верхний, задний, нижний и латеральный. А. Trevisi и соавт. [22] разработали четырехквадрантную классификацию, на основе «метода часов», ранее предложенного L. Moreau и соавт. [23][24], где оценивались межсонные линии, подразделение кавернозной части внутренней сонной артерии на квадранты, и углом между поверхностями сонной артерии и аденомой гипофиза.

При хирургической оценке образований гипофиза на выборке соматотропином Р.В. Плетнев и соавт. определили неблагоприятные интраоперационные характеристики продолженного роста опухоли: багрово-серый цвет солидного компонента, высокая васкуляризация и плотно-эластичная консистенция опухоли [25]. Полученные данные стали доступны благодаря внедрению эндоскопов в транссфеноидальную хирургию [26]: имея панорамный вид анатомической области, нейрохирурги могут напрямую визуализировать медиальную стенку кавернозного синуса и, таким образом, отличать компрессию от инвазии АГ параселлярной области. Кроме того, в опытных руках возможна резекция медиальной стенки и хирургическое удаление мягких аденом в латеральном отделе синуса [17][27–31]. Однако, несмотря на технологический прогресс, определение микроскопической инвазии по-прежнему остается невозможным. В последних работах подчеркиваются возможные преимущества интраоперационной МРТ [32], которая может с большей точностью определять инвазию АГ.

## Гистологический метод

Инвазия твердой мозговой оболочки опухолью гипофиза иногда можно подтвердить гистологически [33][34]. Как правило, в таких случаях определяется высокий пролиферативный индекс опухоли [35][36] и большие размерами образований [35][37][38]. Однако имеются сведения об инвазивных микроаденомах с низким пролиферативным индексом, это позволяет предположить, что инвазивность может определяться не только размерами образования, но и биологическими свойствами [33].

В 1986 г. Selman и соавт. [35] определили, что гистологическая инвазивность определялось чаще (51 из 60 случаев; 85%), чем хирургическая. Противоположные данные были получены в исследовании B. Scheithauer и соавт., где сообщалось о частоте инвазии в 35% среди 365 аденом [33]. Расхождение между исследованиями отражает сложность определения гистологической инвазии, что связано как с вопросами классификации, так и недостаточным объемом ткани для исследования.

Учитывая ограничения представленных методов, на сегодняшний день существует необходимость поиска предикторов течения и биомаркеров инвазивности АГ.

## ПЕРСПЕКТИВЫ изучения БИОЛОГИЧЕСКИХ МАРКЕРОВ инвазивности аденом гипофиза

За последние 20 лет классификация опухолей гипофиза по ВОЗ в рамках Endocrine Tumor classification books (ENDO) пересматривалась трижды: 2004 г. (3‑е издание, ENDOЗ), 2017 г. (4‑е издание, ENDO4) и 2022 г. (5‑е издание, ENDO5) (De Lellis et al. 2004, Lloyd et al. 2017). В Classification of Central Nervous System Tumors (CNS5) и ENDO5 была введена новая номенклатура опухолей аденогипофиза — гипофизарная нейроэндокринная опухоль (ГипНЭО, Pituitary Neuroendocrine Tumors — PitNET) с определением гистологического подтипа в соответствии с классификацией нейроэндокринных неоплазий других органов. Последняя классификация также выделяет категорию аденом гипофиза «высокого риска», в которую включаются подтипы с агрессивным течением: редкогранулированные соматотропиномы, лактотрофные аденомы у мужчин, аденомы из клеток Крука, «молчащие» кортикортрофные аденомы и новый подтип — плюригормональные аденомы с положительной экспрессией PIT-1 (ранее назывались «молчащими» аденомами III подтипа). В связи с агрессивностью течения, пациентов с аденомами «высокого риска» необходимо тщательно наблюдать [2].

Также изменения коснулись наименования «карциномы гипофиза», которое было заменено на метастазирующую ГипНЭО. Основой для классификации и определения морфологического варианта ГипНЭО является иммуногистохимическое определение экспрессии факторов транскрипции гипофиза. Вклад митотической активности и маркеров пролиферации в классификации остается неопределенным как в CNS5, так и в ENDO5, поскольку отсутствуют однозначные отрезные значения данных показателей [39].

В некоторых отношениях агрессивные и инвазивные аденомы гипофиза могут отличаться в клиническом течении, при этом иметь одинаковую молекулярную основу с точки зрения злокачественности и инвазивности (рис. 2).

**Figure:**
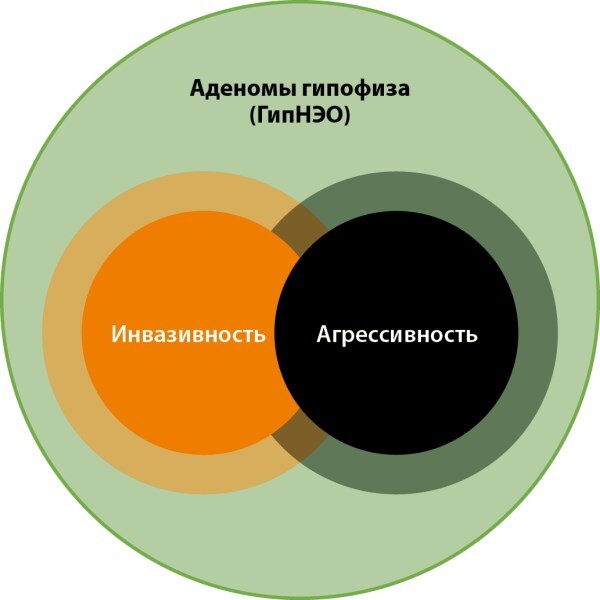
Рисунок 2. Теоретическое представление патофизиологических особенностей АГ. Примечание. Инвазивные и агрессивные АГ по биологическим свойствам очень похожи между собой. Как правило, в клинической практике агрессивная АГ с высоким пролиферативным индексом носит инвазивный характер. Однако не всегда распространяющаяся в кавернозный синус АГ, имеющая признаки инвазии с радиологической точки зрения, находит в этом хирургическое или гистологическое подтверждение. С другой стороны, небольших размеров эндоселлярные АГ могут обладать высоким инвазивным потенциалом.

Белки p53 и Ki-67 являются общими биомаркерами агрессивности различных видов опухолей. Однако интерпретация их экспрессии в аденомах гипофиза зачастую затруднительна, в связи с чем существует потребность в поиске более специфичных маркеров агрессивности и инвазии.

Согласно систематическому обзору 2023 г. [40] выделен ряд экспрессируемых биомаркеров, положительно коррелирующих с инвазивностью и рецидивирующим течением АГ. В соответствии с их ролью в патогенезе выделяют следующие группы биомаркеров с различным механизмом действия:

1) нечувствительность клеток к сигналам, подавляющим рост: минихромосомный поддерживающий белок 7 (MCM-7 protein);

2) ускользание от иммунной системы: циклооксигеназа 2 (COX-2), аргиназа 1 (ARG-1), белок программируемой клеточной смерти 1 (PD-1), лиганд рецептора программируемой клеточной смерти 2 (PD-L2), кластер дифференцировки CD80/CD86;

3) стимуляция опухолевого ангиогенеза: эндотелиальная специфическая молекула 1 (endothelial cell-specific molecule 1 — ESM-1), рецептор фактора роста фибробласта 4 (FGFR4), матричная металлопротеиназа 9 (MMP-9), ген трансформации опухоли гипофиза (PTTG);

4) самообеспечение митогенными сигналами: рецептор эпидермального фактора роста (EGFR);

5) тканевая инвазия: матриксная металлопротеиназа 9 (MMP-9), фасцин.

Также определены маркеры, экспрессия которых обратно коррелирует с инвазивностью и связана с рецидивирующим течением АГ. Данные маркеры распределены по следующим звеньям патогенеза:

1) нечувствительность клеток к сигналам, подавляющим рост: трансформирующий фактор роста β1, белки Smad;

2) стимуляция опухолевого ангиогенеза: тканевой ингибитор металлопротеиназ-1 (ТИМП-1);

3) тканевая инвазия: фактор-ингибитор пути Wnt-1;

4) прочие: ко-экспрессия глиального фибриллярного кислого белка и цитокератина, а также экспрессия эстрогеновых рецепторов α-36 и α-66.

Лиганд рецептора программируемой клеточной смерти 1 (PD-L1), циклин А, цитотоксический связанный с Т-лимфоцитом белок 4 (CTLA-4), белок S100, рецептор эфрина, галектин-3, молекула адгезии нейронов, протеин тирозин фосфатаза 4А3 и фактор стероидогенный-1 не имели значимой связи с рецидивирующим течением и инвазией АГ.

Таким образом, ММП являются одними из биомаркеров-кандидатов. Они не только расщепляют компоненты ВКМ, как коллаген и ламинин, но также участвуют в индуцированном опухолью ангиогенезе и тесно связаны с персистирующим воспалением, играющим важную роль в возникновении и прогрессировании опухолевых заболеваний с инвазивным потенциалом [41].

## Общая характеристика матриксных металлопротеиназ

Матриксные металлопротеиназы (матриксины) — представители суперсемейства мультидоменных внеклеточных цинк-зависимых эндопептидаз (metzincins), контролирующих обмен белковых компонентов ВКМ, мембранных рецепторов, цитокинов, факторов роста и хемокинов. Они принимают участие в пролиферации, миграции (адгезии/дисперсии), дифференцировке, ангиогенезе и иммунном ответе. ММП секретируются клетками соединительной ткани, в частности, фибробластами, эндотелиоцитами, остеобластами, гладкомышечными клетками кровеносных сосудов, макрофагами, лимфоцитами, цитотрофобластами и нейтрофилами [42–44].

Самое раннее описание ММП датируется 1949 г. [45]. В нем были представлены деполимеризирующие ферменты, которые, как предполагалось, могли способствовать росту опухоли, делая строму соединительной ткани и мелкие кровеносные сосуды более «рыхлыми». Спустя 13 лет, Gross J. и Lapierre C. [45] в 1962 г. выделили коллагеназу, отвечающую за резорбцию хвоста головастика. А уже в 1968 г. впервые была идентифицирована коллагеназа (ныне известная как MMП-1) в коже человека [46]. Так как общая классификация ММП на тот момент отсутствовала, многие ферменты «открывались» повторно. Во время конференции "Destin Beach Matrix Metalloproteinase" в 1989 г. Harris Ed. Jr. и его коллеги предложили наименование для данного класса ферментов — «матриксные металлопротеиназы» или «матриксины». Впоследствии Международный союз биохимии и молекулярной биологии присвоил семейству предложенное название, а также назначил каждому представителю порядковый номер [47]. К настоящему времени у позвоночных животных описано 28 ММП и, по меньшей мере, 23 из них экспрессируются в тканях человека [8].

## Строение и биосинтез ММП

Общая структура ММП представлена несколькими доменами: про-доменом, каталитическим доменом, петлевым доменом и гемопексиновым доменом (рис. 3). В каталитическом домене активный центр представлен тремя гистидиновыми остатками, связанными ионом цинком, который, в свою очередь, через консервативный остаток цистеина координирует работу про-домена (так называемый цистеиновый переключатель). MMП-2 и MMП-9 отличаются от других представителей ММП наличием в активном центре еще трех молекул фибронектина II типа, облегчающие связывание эндопептидаз с коллагеном (Bode et al. 1999). C-терминальный гемопексиновый домен участвует как в распознавании субстрата, так и в его деградации за счет четырехлопастной структуры, разрушающей тройную спираль коллагена. Однако данный домен отсутствует у MMП-7, ММП-23 и MMП-26 [42].

**Figure:**
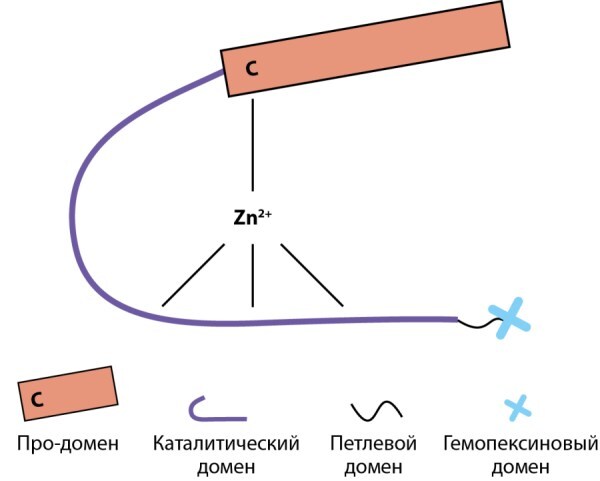
Рисунок 3. Схематическое представление общей структуры ММП.

Биосинтез ММП начинается с молекул-предшественников — пре-проММП — от которых во время трансляции отщепляется сигнальный пептид и образуется неактивная форма фермента — проММП или зимоген. Чтобы зимоген активировался, про-домен молекулы протеолитически удаляется при помощи эндопептидаз (других ММП/сериновых протеаз/плазмина/фурина) от каталитического домена. Данная реакция сопровождается дестабилизацией цистеин-цинкового соединения и образованием активных форм ферментов (рис. 4) [42].

**Figure:**
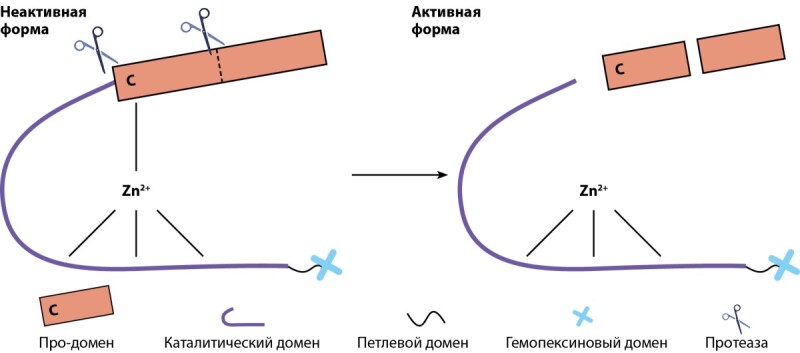
Рисунок 4. Схематическое представление активации ММП.

Для поддержания динамического ремоделирования и обеспечения стабильности ВКМ действие предшественников и активных форм ММП балансируется специфическими ингибиторами металлопротеиназ — тканевыми ингибиторами металлопротеиназ (ТИМП-1, -2, -3 и -4) и неспецифическими ингибиторами протеиназ — ингибиторами α1-протеиназы и α2-макроглобулином [8].

## Классификация ММП

ММП имеет несколько классификаций.

1. В зависимости от доменной структуры фермента: выделяют восемь групп, три из которых фиксированы на мембране клеток, пять других секретируются во ВКМ [48].

2. В зависимости от субстратной специфичности фермента:

- коллагеназы (ММП-1, -8 и -13);

- желатиназы (ММП-2 и -9);

- стромелизины (ММП-3, -10 и -11);

- группа ММП, состоящая из матрилизина (ММП-7), металлоэластазы (ММП-12), эмализина (ММП-20), эндометазы (ММП-26) и эпилизина (ММП-28).

В данной номенклатуре мембранные ММП (ММП-14, -15, -16, -17, -24 и -25) рассматриваются как отдельный класс.

Среди всех ММП при инвазивных аденомах гипофиза наиболее изучены желатиназы (табл. 1) [42]. Очевидная связь данных ферментов с метастазированием опухолей привела к появлению множества научных работ о роли ММП-2 и -9 в различных злокачественных процессах. Для роста опухоли необходимо развитие новой сосудистой системы. Желатиназы, обеспечивая протеолитическую деградацию ламинина-5 и базальной мембраны сосудов, высвобождают VEGF и открывают путь для миграции эндотелиальных клеток, что в совокупности способствует активному процессу ангиогенеза [49]. Эта гипотеза впервые была подтверждена в исследованиях на мышиных моделях с нокаутированным геном MMП-2 и подавлением прогрессирования опухолевого процесса [50]. В связи с этим ММП считаются одними из важнейших участников роста и метастазирования новообразований, что нашло подтверждение в множестве публикаций, посвященных злокачественным заболеваниям.

**Table:** Таблица 1. Типы желатиназ [42] Примечание: ВКМ — внеклеточный матрикс; ИЛ-8 — интерлейкин-8; ИЛ-1b — интерлейкин-1b; ММП-2 — матриксная металлопротеиназа-2; ММП-9 — матриксная металлопротеиназа-9; ФНО-a и -b — фактор некроза опухоли-a и -b; ИНФ-t — интерферон-t.

ММП	Дополнительное название	Субстрат фермента	Продукция фермента	Вызываемые заболевания	Дополнительная информация
2	Желатиназа А	Коллаген I, III, IV, V и VII типов, желатин, некоторые гликопротеины ВКМ, фибронектин, ламинин, аггрекан, эластин, тенасцин, основной белок миелина и витронектин	Клетки: дермальные фибробласты, кератиноциты, эндотелиалиоциты, хондроциты, остеобласты, лейкоциты, тромбоциты и моноциты.	Стимулирование и подавление воспаления, бронхиальная астма?, фиброз, сердечно-сосудистые заболевания и онкологические заболевания	Экспрессия ММП-2 является конститутивной, ФНО-a и -b стимулируют ее продукцию, а ИНФ-t подавляет.
9	Желатиназа B	Коллаген IV, V и XI типов, цитокины, эластин, аггрекан, декорин, ламинин, энтацин, основной белок миелина, казеин, хемокины, ИЛ-8 и ИЛ-1b	Клетки: нейтрофилы, макрофаги, полиморфонуклеарные лейкоциты, остеобласты, эпителиоциты, фибробласты, дендритные клетки, гранулоциты, Т-клетки и кератиноциты.	Сердечно-сосудистые заболевания, воспаление и онкологические заболевания	Выделена вместе с нейтрофилами в 1974 году. ММП-9

## Роль внЕклеточного матрикса и матриксных металлопротеиназ в инвазии аденом гипофиза

Peker et al. [51] исследовали экспрессию коллагена ВКМ в турецком седле. Они первыми обнаружили различия в экспрессии коллагена между капсулой гипофиза и твердой мозговой оболочкой: обе ткани содержат I и II типы коллагена, в то время как III, IV и V обнаруживаются только в капсуле гипофиза. При этом в исследовании Kawamoto et al., где исследовался ВКМ твердой мозговой оболочки [52], коллаген IV типа определен как основной структурный компонент. Авторы предположили, что желатиназы (ММП-2 и -9 типов) связаны с инвазией опухоли. В исследовании Ceylan et al. [53], в отличие от предыдущих работ, медиальная стенка и капсула гипофиза определялись как отдельные структуры, с высокой экспрессией коллагена IV типа.

Несмотря на противоречия в отношении структуры медиальной стенки кавернозного синуса и экспрессии коллагена, все исследования указывают на то, что коллаген IV типа является ключевым компонентом ВКМ гипоталамо-гипофизарной области. Коллаген данного типа подвергается воздействию желатиназ в процессе инвазии аденом гипофиза. Kawamoto et al. [52][54] первыми обнаружили, что при иммуногистохимическом исследовании инвазивных аденом определяется высокая экспрессия MMП-9 по сравнению с неинвазивными. Это исследование впервые определило концепцию патогенеза инвазивного роста аденом гипофиза, опосредованного действием ММП (рис. 5).

**Figure:**
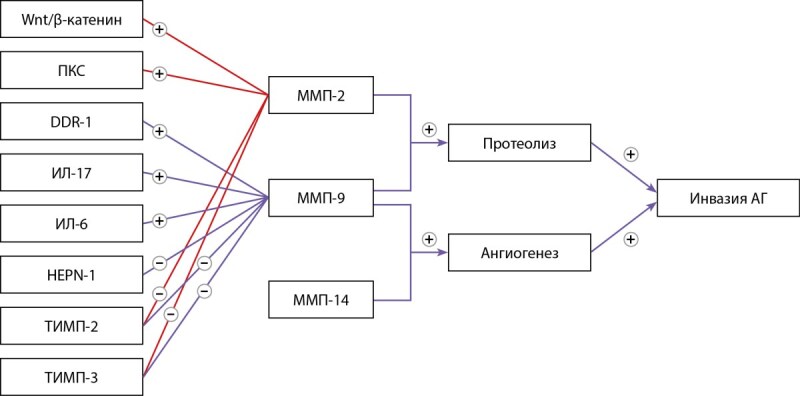
Рисунок 5. Схема регуляции членов семейства MMП при инвазивных аденомах гипофиза [55]. Примечание. Wnt/b-катенин — Wnt/β-catenin signaling pathways; ПКС — протеинкиназа С; DDR-1 — discoidin domain receptor tyrosine kinase-1; ИЛ-17 — интерлейкин-17; ИЛ-6 — интерлейкин-6; HEPN-1 — нepatocellular carcinoma, down-regulated-1; ТИМП-2 — тканевой ингибитор металлопротеиназ-2; ТИМП-3 — тканевой ингибитор металлопротеиназ-3; ММП-2 — матриксная металлопротеиназа-2; ММП-9 — матриксная металлопротеиназа-9; ММП-14 — матриксная металлопротеиназа-14; (+) — стимуляция продукции; (-) — подавление продукции.

ММП-9 — первый представитель, экспрессия которого была повышена в аденомах с инвазией в кавернозный синус [52]. Корреляция между экспрессией MMП-9 и степенью инвазии аденом гипофиза была подтверждена многими исследователями как на образцах опухолей [5][7][56–59], так и на клеточных линиях [60]. Более поздние исследования показали, что экспрессия индуктора внеклеточной матриксной металлопротеиназы (extracellular matrix metalloproteinase inducer, EMMPRIN) [61][62], MMП-14 [63][64], MMП-2 [5][57][65] прямо коррелировали с инвазивностью. При этом для ТИМП-3 [66][67] корреляция между экспрессией и инвазивностью была обратной, как и в случае белка RECK (reversion-inducing cysteine-rich protein with Kazal motifs) [68]. Большинство вышеописанных исследований выполнены на образцах пролактином или аденом со смешанной секрецией. А противоречивые результаты в отношении ТИМП-2 указывают на то, что разные типы гормональной продукции могут иметь различные сигнальные пути в отношении инвазии [59][69][70].

В обзоре Yang Q. et al. 2019 г. обобщены известные молекулярные основы инвазивности аденом гипофиза. Индуцируемый гипоксией фактор-1α (hypoxia-inducible factors, HIF-1α), ген, трансформирующий опухоль гипофиза (pituitary tumor transforming gene, PTTG), фактор роста фибробластов-2 (fibroblast growth factor, FGF-2), фактор роста эндотелия сосудов (vascular endothelial growth factor, VEGF) и MMП (преимущественно ММП-9 и ММП-2) — ключевые молекулы, связанные с распространением аденомы гипофиза в подлежащие структуры. Данные молекулы могут индуцировать клеточную пролиферацию, эпителиально-мезенхимальный переход (ЭМП), деградацию, ангиогенез и ремоделирование ВКМ. HIF-1α, действие которого может индуцироваться гипоксией или апоплексией аденомы, рассматривается в качестве пускового фактора инвазивной трансформации. Дальнейший каскад включает усиление ангиогенеза, опосредованного VEGF, индукцию ЭМП под действием PTTG и деградацию ВКМ за счет ММП. Тем самым создается многокомпонентная интерактивная сеть микроокружения опухоли (рис. 6) [71].

**Figure:**
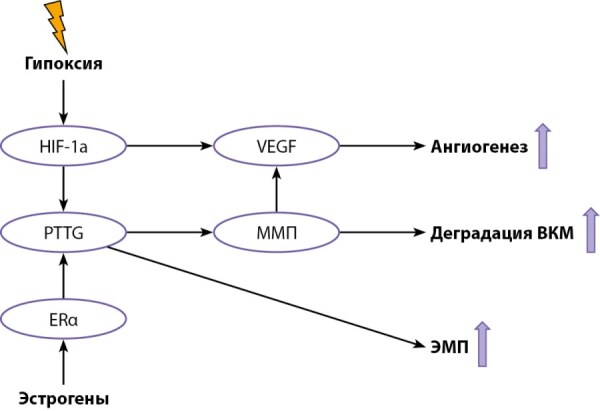
Рисунок 6. Взаимодействие ядерных молекул между собой и их связь с эпителиально-мезенхимальный переходом, ангиогенезом и деградацией ВКМ. Примечание. HIF-1a — hypoxia-inducible factor 1-alpha (фактор, индуцируемый гипоксией 1-альфа); PTTG — pituitary tumor transforming gene; ERα — estrogen receptor alfa (рецепторы эстрогена альфа); VEGF — vascular endothelial growth factor (фактор роста эндотелия сосудов); ММП — матриксная металлопротеиназа.

## Определение концентрации и активности ммп в крови

В настоящее время возможно определять концентрацию и активность ММП при помощи иммуноферментного анализа (ELISA). Циркулирующие ММП активно изучались в различных областях медицины с целью поиска новых биомаркеров. В исследовании Morgia G. et al. приняли участие 40 пациентов с раком предстательной железы (РПЖ), из которых у 20 была диагностирована карцинома, не распространяющаяся за пределы железы, а у других 20 имелись отдаленные метастазы. В контрольную группу вошли 20 пациентов с доброкачественной гиперплазией предстательной железы и 20 здоровых добровольцев. Выявлено значимое повышение концентрации и активности ММП-2, ММП-9 и ММП-13 в плазме у пациентов с РПЖ, особенно при наличии отдаленных метастазов, по сравнению с контрольной группой. Активность и концентрация ММП значимо снижались после радикального лечения РПЖ. Авторы пришли к выводу, что активность ММП в плазме в сочетании с простатспецифичексим антигеном можно использовать для мониторинга терапевтической эффективности у пациентов с распространенной аденокарциномой предстательной железы. Данные других исследований подтверждают эту гипотезу и предполагают, что ММП могут использоваться в качестве маркеров прогрессирования опухолевого процесса [72]. Авторы обращают внимание, что оценку концентрации и активности ММП в плазме всегда следует проводить после исключения сопутствующих заболеваний — ревматоидного артрита, аневризмы аорты, инфаркта миокарда, заболеваний печени или других новообразований, так как данные состояния повышают концентрацию и активность ММП [73].

Многие исследователи не рекомендуют использовать сыворотку крови для исследования, так как коагуляция значимо влияет на результаты анализа, особенно в случае ММП-2, -9 и -13 [73][74]. То же самое относится к ТИМП-1, уровни которого в сыворотке были в пять-семь раз выше, чем в плазме, что, вероятно, связано с высвобождением ферментов во время коагуляции [73].

Тем не менее в исследовании Kasurinen A. et al., включавшем 240 пациентов с раком желудка, обнаружено, что сывороточная MMП-14 служила независимым прогностическим фактором у данной группы больных. Выживаемость была ниже среди пациентов с высоким уровнем MMП-14 (особенно среди мужчин, при стадии pT3-4, наличии отдаленных метастазов). Результаты исследования согласуются с ранее определенной ролью MMП-14 в инвазии опухолевых клеток и метастазировании [75]. Это исследование также предполагает, что высокие уровни ММП-14 в сыворотке крови при раке желудка могут служить маркером прогноза заболевания и указывать на наличие отдаленных метастазов [76].

Guo H. et al. исследовали взаимосвязь инвазии аденом гипофиза и сывороточной концентрации ММП. В группу исследования вошли 58 пациентов с инвазивными АГ и 50 — неинвазивными без уточнения характера гормональной активности. У всех участников исследования были оценены уровни экспрессии ММП-9 и ТИМП-1 как в послеоперационном материале, так и в сыворотке крови, а также проанализирована взаимосвязь показателей ферментов с прогнозом заболевания. Уровни экспрессии MMП-9 и ТИМП-1 в послеоперационном материале измеряли с помощью иммуногистохимического исследования, вестерн-блоттинга и ПЦР с обратной транскрипцией. Значения ММП-9 и ТИМП-1 в сыворотке крови пациентов исследовали посредством ELISA. Уровни сывороточной MMП-9 при инвазивных аденомах гипофиза были значительно выше, чем при неинвазивных. Концентрация ТИМП-1 обратно коррелировала с инвазивностью (p<0,05) (рис. 7). Таким образом, изменения в экспрессии и концентрации MMП-9 и ТИМП-1, вероятно, играют важную роль в инвазии аденом гипофиза и могут служить предикторами прогноза заболевания у данной когорты пациентов [77].

**Figure:**
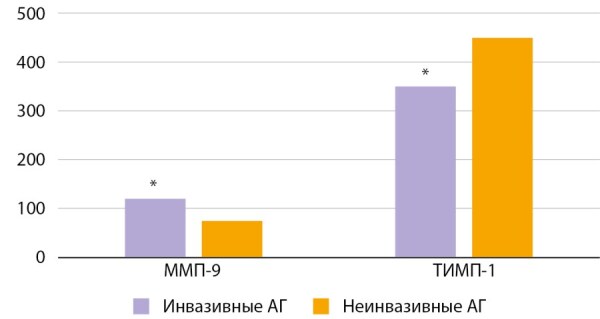
Рисунок 7. Экспрессия сывороточных ММП-9 (матриксная металлопротеиназа-9) и ТИМП-1 (тканевой ингибитор металлопротеиназ-1), нг/мл. Сравнение инвазивных и неинвазивных аденом гипофиза, * p<0,05 [77].

Из представленных исследований следует, что ММП являются перспективными маркерами инвазивности аденом гипофиза. Выбор материала для анализа является важным аспектом, как и четкое разделение групп по гормональной активности и сопоставление с клиническими характеристиками, что необходимо при планировании дальнейших исследований в данной области.

## Перспективы в терапии

С 1990 до начала 2000-х годов синтетические ингибиторы ММП (ИММП) изучались при различных типах рака. Несмотря на многообещающие доклинические данные, ни одно из испытаний не увенчалось успехом — не было снижения опухолевой нагрузки или улучшения общей выживаемости. Кроме того, ИММП вызывали непредвиденные серьезные побочные эффекты: изнурительный скелетно-мышечный синдром, нарушения функции печени и ЖКТ, гематологические заболевания и др.

В настоящее время стало понятно, что некоторые ММП обладают противоопухолевой активностью, поэтому ИММП широкого спектра действия, используемые в первоначальных исследованиях, могли блокировать онкосупрессорные ММП и приводить к прогрессированию злокачественного новообразования. Также ИММП были испытаны на группах пациентов с диссеминированным заболеванием, тогда как роль ММП доказана на ранних стадиях опухолевого роста. В новых исследованиях изучаются селективные ИМПП, что позволит пересмотреть отношение к новому классу препаратов для лечения рака. Предполагается, что новые испытания должны сосредоточиться на ранних стадиях заболевания [78].

В одном исследовании также изучали батимастат — синтетический ингибитор ММП — на крысах линии Fischer 344, с эстроген-индуцированными пролактиномами. Введение препарата способствовало снижению общей массы гипофиза, пролиферации клеток, плотности микрососудов и увеличению апоптотического индекса [79]. При этом III фаза исследования батимастата была прервана в связи с локальной токсичностью, медленным набором пациентов и появлением нового препарата — Маримастата. Однако исследования III фазы по Маримастату (как и по Таномастату, Приномастату, Ребимастату) также были прекращены досрочно из-за выраженных нежелательных явлений [80].

## Заключение

Инвазивные аденомы гипофиза остаются серьезной проблемой, затрудняющей лечение пациентов, что определяет актуальность исследований в данном направлении и необходимость поиска дополнительных инструментов для ранней верификации более агрессивных аденом гипофиза.

Неоднократно предпринимались попытки определения молекулярных механизмов инвазивного роста АГ, однако представления о его патогенезе до сих пор остаются неоднозначны, что определяется вовлеченностью многих молекулярных звеньев и механизмов. Исследования последних двух десятилетий дают предпосылки для расширения понимания инвазивности АГ. HIF-1α, PTTG, FGF-2, VEGF и ММП (в основном ММП-9 и ММП-2) определены в качестве основных молекулярных маркеров, отвечающих за инвазию АГ, благодаря их способности прямо или косвенно индуцировать клеточную пролиферацию, ЭМП, ангиогенез, деградацию и ремоделирование ВМК. ММП изучались и продолжают изучаться в различных областях медицины, в том числе и в онкологии. В литературе представлен широкий спектр исследований, подтверждающих высокую экспрессию ММП-2 и -9 типов в тканях инвазивных АГ. Единичные работы по циркулирующим концентрациям данных ферментов и их ингибиторов указывают на то, что область остается неизученной и перспективной, однако в новых исследованиях необходимо учитывать потенциальные проблемы выбора биологического материала и четкой характеристики исследуемых групп для получения точных результатов.

## Дополнительная информация

Источники финансирования. Работа выполнена по инициативе авторов без привлечения финансирования.

Конфликт интересов. Авторы декларируют отсутствие явных и потенциальных конфликтов интересов, связанных с содержанием настоящей статьи.

Участие авторов. Все авторы одобрили финальную версию статьи перед публикацией, выразили согласие нести ответственность за все аспекты работы, подразумевающую надлежащее изучение и решение вопросов, связанных с точностью или добросовестностью любой части работы.
